# Supplementary material for: Ribonucleic Acid Sequencing Reveals the Upregulation and Resolution of Inflammation and Extracellular Matrix Remodeling in Lidocaine-Treated Human Acute Monocytic Leukemia Cell Line THP-1
Source: Biomedicines. 2024 Feb 23;12(3):509. doi: 10.3390/biomedicines12030509 (PMC10967704; doi:10.3390/biomedicines12030509)
Supplement: Supplementary file 1 [file biomedicines-12-00509-s001.zip › biomedicines-2886349-supplementary.pdf]

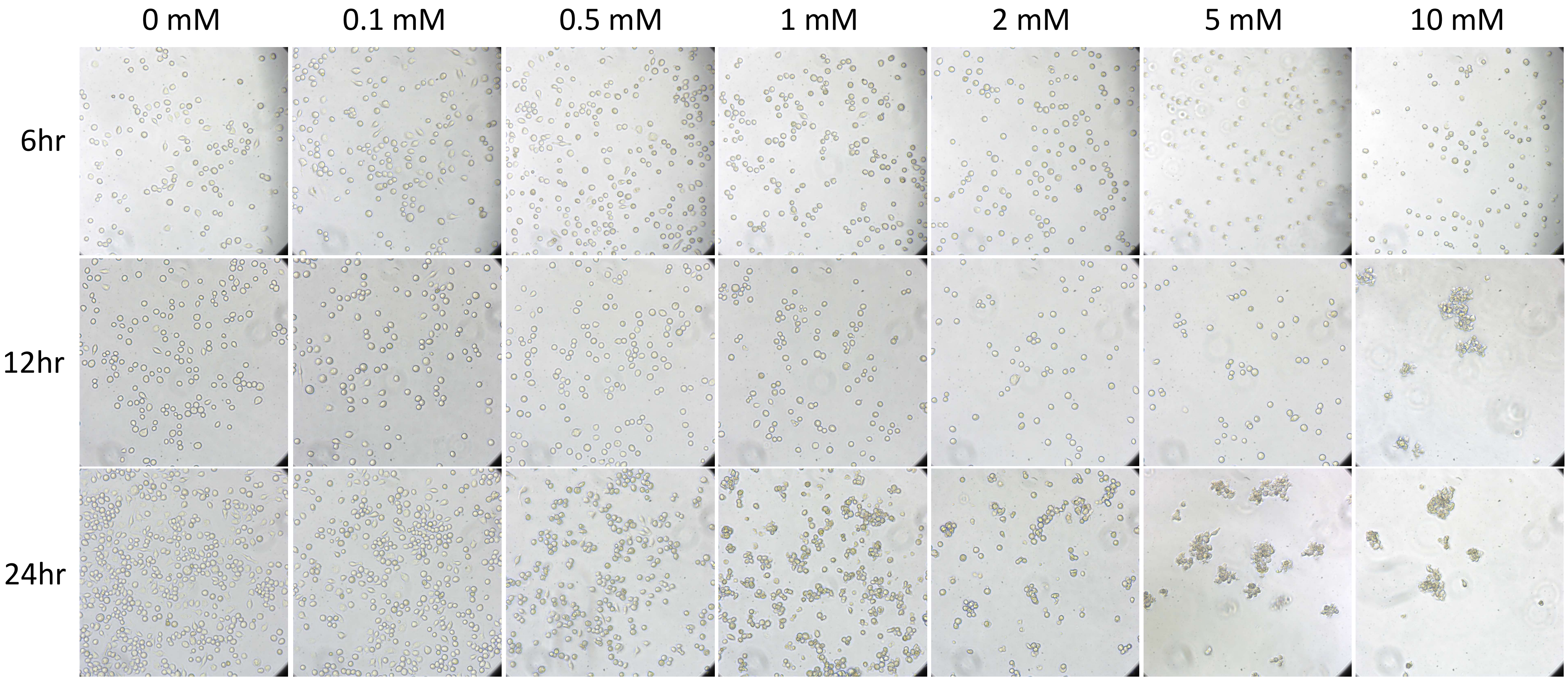

Figure S1. Raw images of bright-field microscopy in untreated and lidocaine-treated THP-1 cells. Cell morphology visualized using light microscope (20x magnification) at various lidocaine concentrations for 0, 6, 12 and 24 h. Representative images (cropped) were shown in Fig. 1B in the main figure.

Figure S2. Protein-protein interactions of identified genes. The STRING protein database was utilized to analyze the protein-protein interactions of GDF15, FGF7, HGF, COL4A3, COL8A2, LAMB2, LAMC2, PDGFRA, and VEGFA.

STRING parameters used to generate this figure.

- full STRING network

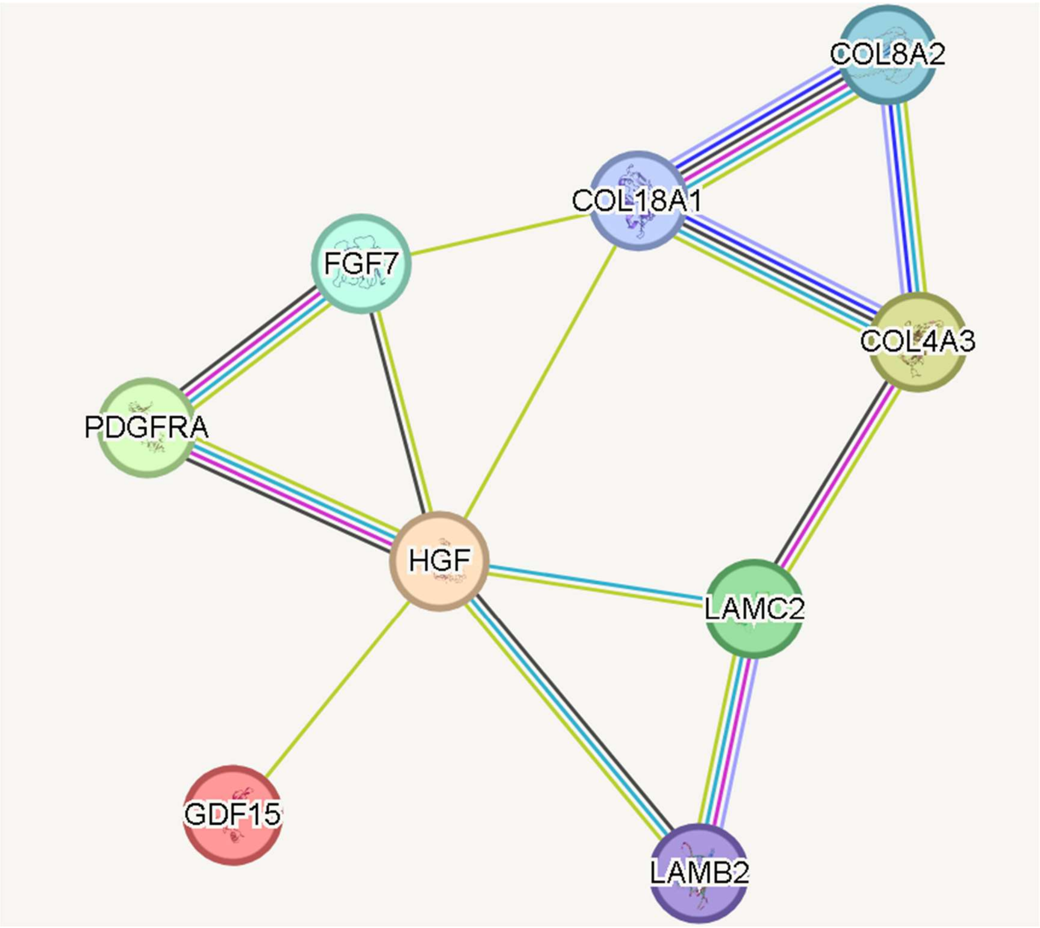

- evidence, line color indicates the type of interaction evidence
- medium confidence 0.400
- number of nodes: 9
- number of edges: 13
- average node degree: 2.89
- avg. local clustering coefficient: 0.652
- expected number of edges: 1
- PPI enrichment p-value: 1.93e-11

Biological Process (Gene Ontology)

GO-term	description	count in network	strength	false discovery rate
GO:0060665	Regulation of branching involved in salivary gland morphog…	2 of 3	3.16	0.0033
GO:0051450	Myoblast proliferation	2 of 7	2.8	0.0075
GO:0072224	Metanephric glomerulus development	2 of 11	2.6	0.0126
GO:0032836	Glomerular basement membrane development	2 of 11	2.6	0.0126
GO:0032835	Glomerulus development	3 of 56	2.07	0.0033
GO:0030198	Extracellular matrix organization	5 of 278	1.6	0.0011
GO:0050673	Epithelial cell proliferation	3 of 170	1.59	0.0312
GO:0007169	Transmembrane receptor protein tyrosine kinase signaling …	5 of 425	1.41	0.0022
GO:0006935	Chemotaxis	5 of 516	1.33	0.0033
GO:0071363	Cellular response to growth factor stimulus	4 of 473	1.27	0.0253
GO:0030335	Positive regulation of cell migration	4 of 529	1.22	0.0335
GO:0007155	Cell adhesion	6 of 965	1.13	0.0033
GO:0009887	Animal organ morphogenesis	6 of 997	1.12	0.0033
GO:0009888	Tissue development	6 of 1723	0.88	0.0214
GO:0009653	Anatomical structure morphogenesis	7 of 2229	0.84	0.0075
GO:0048513	Animal organ development	8 of 3246	0.73	0.0055
GO:0048731	System development	8 of 3867	0.66	0.0129
GO:0032501	Multicellular organismal process	9 of 6490	0.48	0.0288

Molecular Function (Gene Ontology)

GO-term	description	count in network	strength	false discovery rate
GO:0005201	Extracellular matrix structural constituent	4 of 131	1.82	0.0013
GO:0005102	Signaling receptor binding	7 of 1499	1.01	0.0013

Cellular Component (Gene Ontology)

GO-term	description	count in network	strength	false discovery rate
GO:0043256	Laminin complex	2 of 12	2.56	0.0043
GO:0005604	Basement membrane	5 of 98	2.05	8.97e-07
GO:0005581	Collagen trimer	3 of 95	1.84	0.0029
GO:0005788	Endoplasmic reticulum lumen	4 of 312	1.45	0.0026
GO:0005615	Extracellular space	8 of 3247	0.73	0.0017
GO:0012505	Endomembrane system	8 of 4721	0.57	0.0159

Local network cluster (STRING)

cluster	description	count in network	strength	false discovery rate
CL:19651	Laminin interactions	2 of 21	2.32	0.0214
CL:17332	Activated point mutants of FGFR2, and Fibroblast growth fa…	2 of 25	2.24	0.0269
CL:17331	Mixed, incl. FGFR2 ligand binding and activation, and PDGF/…	3 of 45	2.16	0.0011
CL:19458	Collagen formation, and Protein complex involved in cell ad…	4 of 119	1.87	0.00081
CL:17327	Mixed, incl. Constitutive Signaling by Aberrant PI3K in Canc…	4 of 154	1.75	0.0011

KEGG Pathways

pathway	description	count in network	strength	false discovery rate
hsa05218	Melanoma	3 of 72	1.96	0.00037
hsa04512	ECM-receptor interaction	3 of 88	1.87	0.00053
hsa05222	Small cell lung cancer	3 of 92	1.85	0.00053
hsa04974	Protein digestion and absorption	3 of 100	1.82	0.00055
hsa05146	Amoebiasis	3 of 101	1.81	0.00055
hsa04510	Focal adhesion	5 of 195	1.75	2.10e-06
hsa01521	EGFR tyrosine kinase inhibitor resistance	2 of 77	1.75	0.0145
hsa05145	Toxoplasmosis	2 of 103	1.63	0.0237
hsa04151	PI3K-Akt signaling pathway	6 of 349	1.58	8.83e-07
hsa04015	Rap1 signaling pathway	3 of 201	1.51	0.0033
hsa05226	Gastric cancer	2 of 146	1.48	0.0436
hsa04014	Ras signaling pathway	3 of 225	1.47	0.0041
hsa05200	Pathways in cancer	6 of 515	1.41	2.92e-06
hsa04010	MAPK signaling pathway	3 of 286	1.36	0.0075
hsa05165	Human papillomavirus infection	3 of 324	1.31	0.0099
